# Tools for fairness: Increased structure in the selection process reduces discrimination

**DOI:** 10.1371/journal.pone.0189512

**Published:** 2017-12-11

**Authors:** Sima Wolgast, Martin Bäckström, Fredrik Björklund

**Affiliations:** Department of Psychology, Lund University, Lund, Sweden; Charles P. Darby Children’s Research Institute, 173 Ashley Avenue, Charleston, SC 29425, USA, UNITED STATES

## Abstract

Employment discrimination causes problems at the labor market, and is hard to combat. Can increasing the degree of structure when selecting applicants increase fairness? Students were asked to perform a computerized selection task and were either provided with tools for systematizing information about the applicants (structured selection) or no such tools (unstructured selection). We hypothesized and found that a structured process, where employing recruitment tools rather than the recruiter’s impressionistic judgment is key, improves the ability to identify job-relevant criteria and hence selecting more qualified applicants, even when in-group favoritism is tempting (e.g. when the outgroup applicants are more competent). Increasing structure helped recruiters select more competent applicants and reduced ethnic discrimination. Increasing the motivation to carefully follow the structured procedure strengthened these effects further. We conclude that structure pays off, and that motivational factors should be taken into account in order for it to have the optimal effect.

## Introduction

This research seeks to identify means for optimizing selection and counteracting discrimination. While several papers have appeared independently on either the subject of labor discrimination or on the subject of structured procedures in personnel selection, to our knowledge none has investigated whether increasing structure decreases discriminatory behavior when selecting applicants (i.e. not simply providing lower ratings, but actually failing to choose the most qualified applicants). We reports on two laboratory experiments investigating whether relying more on structured procedures in the selection process can make recruiters’ decisions less biased.

### Discrimination in recruitment and selection

Despite indications that there has been a decline in racist attitudes, differential treatment based on ethnicity continues to be a problem in employment decisions [1]. Decision makers discriminate in favor of applicants of their own ethnic group [2, 3] and ethnic minorities are often subject to discrimination in hiring [4, 5, 6].

A common way of studying the prevalence of discrimination in the recruitment process has been field experiments, most notably using correspondence testing, where fictitious job applications are sent to real job-openings. The fictitious applicants have either native-sounding names or foreign-sounding names, and the researchers compare the call back rate between immigrants and natives. Using this method, ethnic discrimination has been detected in several countries in Europe [7, 8, 9], in the United States [3, 5, 10, 11], in Canada [12, 6] and Australia [13]. Hence, the evidence of discrimination on the labor market is remarkably solid.

The current study concerns how discrimination can be counteracted by altering the procedure the recruiter uses. We focus on discrimination in selection rather than recruitment, i.e. on the process of deciding whom to select among an existing group of applicants, a process which is susceptible to bias when there are immigrants among the applicants.

### Stereotyping, biases and discrimination

Selection situations where immigrants are involved are susceptible to bias. For example, mere categorization can create negative bias toward an outgroup, promote in-group favoritism, and result in exclusion of outgroup members [14].

Furthermore, perceived interference with the dominant groups’ goals and competition for resources such as jobs may promote a more active discrimination against members of the outgroup, when compared to discrimination based on implicit attitudes and mere categorization [15]. The amount of information available regarding the applicants influences outcomes too. In low information conditions, decision makers rely more on stereotypes [16, 17]. The present study extends the previous literature in that it investigates whether structured procedures counteract discrimination. What is being manipulated is not the amount of job-relevant information, but rather how the selection process is set up and conducted.

### Structured procedures and biases in personnel selection

There is a consensus among researches that we should strive for structured procedures in recruitment and selection [18, 19, 20, 21, 22, 23]. This is clear not least in the Principles for the validation and use of personnel selection procedures from the Society for Industrial and Organizational Psychology (SIOP, division 14 of the American Psychological Association), and ISO-standard 10667–1:2011 Assessment service delivery—Procedures and methods to assess people in work and organizational settings, which are broadly acknowledged documents with guidance on how to conduct an optimal selection process. Structured procedures should encompass: defining specific criteria related to job content by means of a job-analysis, gathering and evaluating information (options when choosing and conducting selection instruments, interview, evaluating applications and inquiring for references) and decision making.

Despite extensive evidence that structured forms of selection are more valid, recruiters more frequently use the more intuitive, impressionistic and unstructured forms [24, 25, 22, 26, 27, 28]. This allows for biases and subjective preferences to influence the process [20] and thereby increases the risk of discrimination on the labor market. Conversely, a more structured process leads to less biased performance- and hireability assessment of applicants who are overweight [29] or pregnant [30]. It should be noted however that these studies do not concern ethnicity, nor decisions where some applicants are selected and others are not. Thus, although the existing research indicates that structured selection produces more valid selection decisions, there is a lack of research on the effect of structured procedures on selection discrimination. Nevertheless, since previous research has shown that structure reduces bias [19, 20, 31, 16, 30, 29], it appears reasonable to predict that structure should decrease selection discrimination too. Our aim with the present research is to investigate experimentally whether employing a structured procedure (by means of tools for rating and ranking applicants) reduces discrimination against outgroup members. In doing this, we will not study judgements of hireability, which is standard in social psychology research, but rather selection decision behavior. This is important, since discrimination per definition concerns explicit behavior (such as exclusion) whereas judgments and assessments do not.

There are different ways to increase systematicity in the selection process. In the current research, we will provide some participants with a tool related to job-analysis as a way of increasing systematicity. Job-analysis is a broad term for procedures for examining, documenting, and making inferences about work activities, worker attributes, and work contexts, in order to identify relevant criteria and characteristics for a particular job [32]. The job-analysis tool in the present study focuses on the tasks, skills, and characteristics needed to manage the specific job. It helps the recruiter specify relevant tasks and duties, as well as the characteristics needed to achieve them. This should be useful since it helps the recruiter to identify relevant skills, knowledge and abilities possessed by job-applicants, and decrease the risk of relying on idiosyncratic beliefs about job requirements or the recruiters’ own personality traits and attitudes [19, 31]. In sum, job-analysis is a way of decreasing the recruiters’ reliance on preexisting fixed categories when processing information about the applicants, automatic processing which is known to increase the risk of stereotyping and discrimination [16]. Instead, the information processing is more controlled and hence possibly less biased. In the current study, participants in the systematic condition will be working actively with the contents of the job (by means of the job analysis tool) and the CV-reading, which should increase the availability and accessibility of the job-relevant criteria.

Personnel selection involves large amounts of information and puts high demands of the recruiter’s information processing capabilities. In order to help systematize the outcome from the processing of the information regarding the applicants, some participants in the current study will (in addition to the job-analysis tool) be provided with a tool for summing their judgments of individual target persons. Together, the job analysis tool and the calculation tool should lead to less bias related to e.g. applicant’s ethnic group belonging and thereby reduce discrimination.

Arguably, structured procedures should facilitate fair selection. To investigate whether this is so, we experimentally manipulate structure and investigate how this influences selection outcome. We predict that there will be a difference in job-applicant preferences between those who work structured (experimental condition) and those who do not (control condition). We used a fictive job setting where male job-applicants, both Swedes (in-group) and immigrants from the Middle East (a discriminated group on the labor market; [9]), applied for a sales manager position.

Furthermore, the average competence level of the in-group and outgroup differed from each other, allowing for investigation of whether the participants reacted differently depending on whether in-group members or outgroup members were the most competent, and if structured selection has similar effects under these different conditions. The unequally distributed competence level across in-group and outgroup applicants circumvents the reactance effects that often appear in discrimination studies. Participants who are motivated to control their biases tend to overrate target persons that belong to the outgroup [33]. With the current design, which allowed for selecting both in-group and outgroup applicants for the same job opening, the cues of possible bias are weaker than if only one applicant was to be selected or if competence was equally distributed across applicants.

## Study 1

Study 1 had a 2 (structured or unstructured) x 2 (competence: outgroup or ingroup) between group design. In the structured condition participants were aided by tools when selecting job applicants, which was not provided in the unstructured condition. We also manipulated applicant competence, where either the ingroup applicant were the most competent or the outgroup were the most competent. We expected that participants in the conditions where a structured procedure was employed would select competent applicants to a higher degree than participants in the control conditions. In the control conditions, where no tools for structure were available, we expected participants’ selection decisions to be influenced by the processes described in the introduction, and hence select applicants with less actual competence and generally disfavor outgroup applicants. Accordingly, study 1 was designed to test the following main hypothesis:

There should be an interaction between degree of structure and competence (in-group most competent vs outgroup most competent). Compared to working with an unstructured procedure, working with a structured procedure should lead to selecting more outgroup applicants when they are the most competent and fewer outgroup applicants when they are not.

## Materials and methods

### Ethics statement

The studies in this report were approved by the Regional Ethical Review Board in Lund (EPN; Lund, D.nr. 2009–3). Participants received verbal and written information about the study before signing when consenting to participate.

### Participants

Altogether 249 participants, 121 men and 128 women, were included in study 1. They were all Caucasian Swedish students at Lund University. The average age was 23.5 years (*SD* = 3.3).

### Design

We used a 2 (structure or unstructured) X 2 (competence: outgroup or ingroup) between group design. We used two dependent variables: proportion of outgroup applicants selected and average competence of the selected applicants.

### Computer application

A computer application was designed to create a fictive personnel selection setting where the participants acted as recruiters. There were two different conditions. Participants in the structured condition read a work description, and responded to questions about the content of the job. The idea was to mimic a structured recruitment process (with a job-analysis). There were 32 questions, half of which were job-relevant and half job-irrelevant. To make sure that they processed the job-description sufficiently, participants had to spend at least nine minutes reading it and responding to the questions. Participants in the unstructured condition were not provided with any tool for job-analysis, but only with a job-description.

The two conditions also differed in whether or not participants were provided with tools to systemize the information about the candidates. In the structured condition, they were provided with a rating and calculation tool. While reading the CV résumés (see below) they had to rate to what extent they thought the candidates fitted with the job-description. They were also helped calculating sum scores for each applicant, by the computer application, to simplify comparison between applicants. In the unstructured condition, they only read the CV résumés and were not provided with this tool. Participants in the structured conditions had the opportunity to make selection decisions based on explicit job-relevant criteria, whereas participants in the unstructured conditions lacked tools to make these comparisons.

The other factor that was manipulated was the level of competence of applicants. There were two levels: in-group more competent or out-group more competent. High competence and low competence CV résumés were unequally distributed over in-group and out-group applicants. In the first condition the in-group applicants were more competent, the eight ingroup applicants (Swedes) all had high or average competence, whereas the 4 out-group (Middle East) applicants had low competence. In the other condition, instead, out-group applicants were more competent, where the 4 out-group candidates had high competence and the 8 in-group applicants had average or low competence.

### Job-applicants and their résumés

The applicants were presented with CV résumés with information pertaining to their education, past experience and recommendations from managers from former workplaces. The résumés were constructed in relation to six relevant (e.g. establish and maintain interpersonal relationships) and six less relevant (e.g. training and educating others) criteria for the sales manager position, as specified by O*NET. Each résumé belonged to one of three different competence levels (high, average or low). To construct the levels we created a large number of sentences describing applicant competences. These were deliberately created to differ in relevance to the job but also in relation to the level of competence that was depicted. The sentences were rated by a group of students who had read the work description, both on level of competence and relevance, and based on these ratings they were categorized into nine groups, from high competence and high relevance, to low competence and low relevance. To make a résumé of a high level applicant, we selected two sentences with high competence and high relevance, two with average level of competence and average relevance, and two with low level of competence and low relevance. In other words, a high-level applicant was more competent on relevant criteria. A low-level applicant was created by selecting two sentences with high competence and low relevance, two with average level of competence and average relevance, and two with low competence and high relevance. The middle group had a combination of high and low relevance combined with high and low competence such that their total competence was in between the low and high-level applicants. In this way, all applicants appeared to have about the same level of general competence, but in relation to the job the high-level applicants were more competent on the relevant criteria.

The origin of the applicants (in-group or out-group) was signalled by means of photographs. The study included 8 in-group (Swedes) and 4 outgroup (from the Middle East) applicants, all were males around age thirty. The photographs were evaluated by 50 students to be equally attractive.

### Procedure

In the lab, an assistant introduced to the procedure to the participants. The task was to select the four applicants that they judged to be most qualified for the job. Participants were randomly assigned to either the structured or the unstructured condition, and the computer application guided them through the recruiting task.

#### Structured condition

In the structured condition, three modules were presented:

The first module introduced a job-description, listing the central tasks and the key required abilities for the job.
The second module introduced the tool for job-analysis, where the task was to rate how important each kind of content was in relation to the job-description. This produced a list of competence criteria, to be used when choosing applicants.
The third module introduced the 12 applicants. Participants clicked on each photograph to read the corresponding résumé and assess the applicants’ qualifications (0–100) with regard to the competence criteria from the job-analysis. The mean rating of each applicant was shown on the screen. Finally, participants were asked to select the four applicants that they believed to be the most competent, and rank-order them.

#### Unstructured condition

In the unstructured condition there was no tool for job-analysis. The following modules were presented:

The first module introduced the same job-description as module 1 in the structured condition.
The second module was the same as module three in the structured condition, but lacked the rating tool

### Statistical analysis

The hypothesis was tested with factorial ANOVA, since we were interested in the interaction between structure of the recruitment and the level of competence of the in-group and outgroup. The interaction should reveal if a systematic recruitment leads to a fairer selection of applicants, i.e. that the participants in the systematic group are less influenced by applicants origin in comparison with the less systematic group.

Two dependent variables were used to test the hypothesis. The proportion of selected outgroup applicants provides a direct estimate of whether there was an influence from the ingroup out-group competence factor on the selection. The expected number of selected outgroup applicants is .33 because of the unequal number of in-group and out-group applicants. The quality of the selected résumés is the second dependent variable and will indicate if the participants’ performance was affected by the fact that the competence of the in-group and the outgroup differed.

## Results and discussion

**Fig 1:**
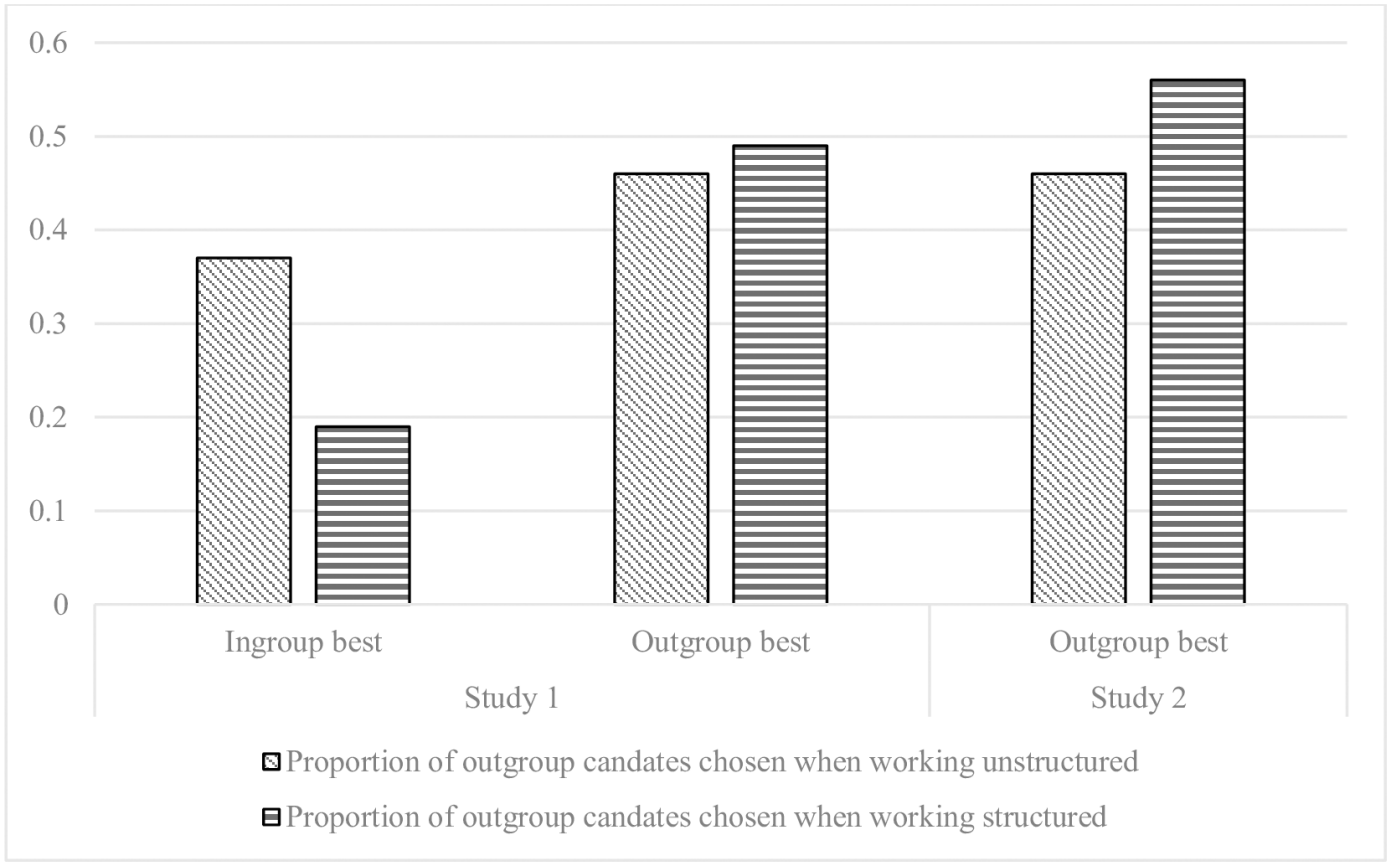
Proportion of outgroup candidates selected in the different conditions.

The proportion of outgroup applicants selected was tested in a factorial ANOVA with competence (in-group or outgroup most competent) and structure (structured or unstructured procedure) as factors and proportion of outgroup applicants selected as dependent variable. Here we expected a significant main effect of competence and a significant interaction effect of structure and competence. As expected, the analysis revealed a strong main effect of competence, *F*(1, 245) = 82.1, *p* < .001, partial η^2^ = .25, indicating that the participants selected more outgroup applicants when the outgroup was the most competent (*M* = .47, *SD* = .17), compared to when the in-group was the most competent (*M* = .28, *SD* = .18), see the left panel of Fig 1. As hypothesized, the ANOVA also indicated a significant interaction effect between structure and competence, *F*(1, 245) = 25.0, *p* < .001, partial η^2^ = .09. Simple effect analyses revealed that, compared to participants in the unstructured condition, participants in the structured condition (as expected) selected fewer outgroup applicants when the in-group was the most competent (*M*_*Diff*_ = -.18, *p* < .001), but—contrary to our expectations—they did not select a significantly higher proportion of outgroup applicants when the outgroup was the most competent (*M*_*Diff*_ = .03, *p* > 0.05).

Concerning average quality of the selected résumés, we expected a main effect of structure (participants in the structured condition would chose applicants of higher competence) as well as an interaction effect between structure and competence (that the difference between the average quality of the selected résumés would be larger when the outgroup applicants were the most competent compared to when the in-group applicants were the most competent). As expected, the results from the performed analysis indicated a significant main effect of structure, *F*(1,245) = 31.17, *p* < .001, with a large effect size, partial η^2^ = 0.71, where participants in the structured conditions generally selected higher quality applicants (*M* = 3.31, *SD* = 0.32) than participants in the unstructured conditions (*M* = 3.09, SD = 0.32). This suggests that structured selection increases the chances of finding the high quality applicants. Contrary to our expectations however, the interaction between structure and competence was non-significant, *F*(1, 245) = 0.012, *p* > .05, but rather there was a significant main effect of competence, *F*(1,245) = 19.68, *p* < .001, *d* = 0.52, indicating that the average quality of the selected résumés was higher when the in-group was best (*M* = 3.29, *SD* = 0.32) compared to when the outgroup was best (*M* = 3.12, *SD* = 0.34) in both the structured and unstructured conditions.

Thus, in sum, study 1 provided partial support for our hypothesis in that participants working with the structured procedure were better at identifying and selecting applicants of higher quality. Contrary to our expectations however, we found no support for the assumption that working with a structured procedure leads to less discrimination of applicants from the outgroup, since participants in the structured condition did not select more outgroup applicants when they were the most competent compared to participants in the unstructured condition. Instead, in both the structured and unstructured conditions there was a tendency to select applicants of less quality when the outgroup was the most competent, thus favoring the in-group applicants. There are several possible explanations to these results:

In study 1 we used 12 résumés where four were of “average competence”. By doing this we created a not so clearly differentiated set of applicant-résumés, since it was more difficult to distinguish the most competent applicants from the average ones than from the low competence ones. The difficulty to distinguish the most competent applicants from the rest of the applicants could explain why participants in the structured conditions, despite working with a structured procedure did not perform better than the unstructured group. This effect may in part be due to information overload causing participants to make their choices based on stereotypes, instead of on data driven processing strategies [16, 17]. In the present study, it might be the case that, when having difficulties processing all information, participants instead relied on their attitudes about the outgroup. Additionally, it should be noted that all résumés of average quality were paired with in-group applicants (there were always eight in-group and four outgroup applicants). This created an asymmetry between the conditions where the in-group applicants were the most competent compared to when the outgroup applicants were the most competent that may partially explain the results when it comes to proportion of outgroup applicants selected: Difficulties to differentiate the most competent applicants from those of average competence always led to the selection of more in-group applicants. In the conditions where the outgroup was the most competent, selection of average quality applicants necessarily led to selection of applicants of the “wrong” ethnicity, whereas the same selection pattern in the conditions where the ingroup applicants were the most competent resulted in the selection of applicants of the “right” ethnicity. It should be noted however, that this can only partially explain the results, since the average quality of the selected résumés was higher when the in-group was best compared to when the outgroup was best in both the structured and unstructured conditions.

Follow-up analyses revealed that one obvious difference between the structured and the unstructured conditions was the time they spent on the task. Almost all participants in the structured condition spent longer time reading and processing the CV résumés than the participants in the unstructured condition (mean times per CV was 109 (*SD* = 37) sec. vs 57 (*SD* = 24) sec.). Can the time spent on reading CVs contribute to explain the success of the participants in performing the selection task? It was found that a significant association between time spent on the task and performance was present only in the structured group when the out-group was best. The correlation was *r* = .35, *p* = .005 between time spent reading CVs and the quality of the selected applicants, and *r* = .36, *p* = .004 between time spent reading CV and rating of the four best applicants. There was also a close to significant correlation between time spent reading CVs and the quality of the selected CV in the unstructured condition when the outgroup was best, *r* = .245, *p* = .057. This suggests that, at least in some situations, those who spent more time performed better, which in turn might reflect how motivated they were to perform the task. This hypothesis will be tested in study 2. The correlation between CV reading time and selection performance can be taken to suggest that those who put time and effort into the task perform better than those who care less about their performance. Introducing a manipulation that provides a response cost to working carelessly should make recruiters more prone to increase their effort to go about the selection task in the intended way. This was tested in study 2. Additionally, since the structured procedure in study 1 had the expected effect when the in-group was best, in study 2, we only used the stimulus material where the outgroup was best (and where the structured procedure did not work in study 1).

## Study 2

The structured procedure did not lead to the selection of more outgroup applicants in study 1. In study 2 we attempted to influence participants’ behaviors in the selection context by providing a new piece of instruction. Drawing on the idea that a behavior can be controlled by antecedents, when a relationship between the behavior and a consequence is described [34, 35], we introduced an instruction which informed the participants that if they did not involve themselves enough in the selection procedure there would be consequences in the form of a response cost (they would have to do it all over again).

Increasing the motivation to carry out the selection task carefully should leave less room for individual differences and thereby strengthen the effect of the experimental manipulation. In related research, increasing the accountability of raters does indeed increase the accuracy in performance appraisal tasks, through increased attentiveness and notetaking [36]. Hence, in study 2 we tested whether enhancing the motivation to carry out the task carefully increases the effect of working with a structured procedure, in comparison to what was found in study 1. As in study 1, we expected that working with the structured procedure would lead to less discrimination (i.e. selecting comparatively more outgroup applicants when they are the most competent). Given that the focus of study 2 is the condition where the outgroup applicants are the most competent, this was the only condition used in study 2 (i.e. there were no conditions where the in-group applicants were best).

## Method and materials

### Participants

There were 104 participants (51 male and 53 female). All participants were Caucasian Swedish students at Lund University. The average age of the participants was 23.8 years (*SD* = 3.3).

### Design

Study 2 had a between-group design with two groups. Participants were randomly assigned to either the experimental condition or to the control condition (where no tools were provided).

### Materials

The same computer application, photographs of job-applicants, and CV résumés were used as in the part of study 1 where the outgroup applicants were the most competent. Thus, the four outgroup applicants had résumés indicating high competence and the four in-group applicants had résumés indicating average competence. Prior to reading the CV résumés participants in both conditions were presented to a “response cost” manipulation:

“Your next task takes at least 30 minutes. It is important not to be careless when working on it. For the results of your effort to be useful, you need to reach a certain level of performance. If you are careless and do not reach a satisfactory level, you will unfortunately have to do the complete task again.”

## Results and discussion

The results supported our hypothesis that the proportion of outgroup applicants selected would be higher in the structured condition (*M* = .56, *SD* = 0.18) than in the unstructured (*M* = .46, *SD* = 0.18); *F*(1, 101) = 7.94, *p* = .006, *d* = 0.56, see right panel of Fig 1. In addition, as in the previous study, participants who worked with a structured procedure (*M* = 3.47, *SD* = 0.27) selected applicants with a higher mean quality compared to those who did not (*M* = 3.24, *SD* = 0.33); *F*(1, 101) = 15,51, *p* < .001, *d* = 0.77).

In the previous study, we found that participants in the experimental condition spent more time on the task, and that the more time they spent the better they performed. Also in the control group, there was a tendency for participants who spent more time to perform better. In this study, the time spent on reading the résumés and making the decisions was longer, *M* = 78 (*SD* = 39) and *M* = 137 (*SD* = 49) for the control and experiment group, respectively. This suggests that the motivation to work thoroughly was stronger after the response cost manipulation. Notably, the correlation between time and performance was non-significant (*p* > .05) in both groups. To check whether the manipulation really influenced the participants to do a better job when selecting the applicants, we compared the correlations between the ratings of the applicants and the final choices. If the participants in the experimental group did a better job when rating résumés, then the correlation should be stronger between choice and ratings. In study 1, the correlation was *r*(1498) = .358, *p* < .001, suggesting that selected applicants were ranked higher (*N* is based on the 12 rated résumés, not on participants). In fact, this correlation was somewhat higher when the out-group was best, *r*(742) = .408, *p* < .001 compared with *r*(754) = .306, *p* < .001. In study 2, the correlation was *r*(610) = .521, *p* < .001. These three correlations were found to differ significantly from one another, using *z*-tests (*z* = 2.26, *p* = .020, *z* = 4.77, *p* < .001, and *z* = 2.64, *p* = .008).

Thus, in comparison to study 1, where no significant differences between the experiment group and control group were found when the outgroup applicants were the most competent, our “response cost” manipulation appeared to induce a change in performance and increase the effect of the structured procedure. The results support the general hypothesis that enhanced motivation to perform selection tasks carefully increases the effect of a structured procedure.

## General discussion

Our studies aimed at experimentally investigating the possible benefits of a structured procedure in selection as a means to counteract discrimination. In the following sections, we discuss the major contributions of the performed studies, as well their central limitations and some recommendations for future research.

### Theoretical and empirical contributions

Several studies have already validated the general benefits of working with structured procedures when recruiting [22], but to our knowledge none have studied the effects of structured procedures on discrimination experimentally. The experimental design of our studies allows for causal inferences about the effects of the structured procedure in a selection context, hence providing an important addition to a field where the emphasis has been on ecologically valid yet correlational research. Furthermore, our studies employed a novel method for conducting recruitment experiments, based on a computer application. It was designed to resemble an actual selection situation but also enabled structuring information and recording the behavior of the participants, which are clear advantages in comparison to more traditional (e.g. paper-and pencil) methods.

Regarding findings, the main contribution is that they provide experimental support for the hypotheses that increasing the degree of structure leads to higher quality in selection decisions. The strongest effect was for average quality of the selected résumés. Working with the structured procedure lead to an improved ability to select more competent applicants in both studies. The results regarding counteracting discrimination were somewhat more mixed and warrant further discussion.

In our first study of the effects of structured procedures on outgroup discrimination (Study 1), we found an effect on average quality of the selected résumés, but—contrary to our hypothesis—no effect on discrimination (i.e. proportion of outgroup applicants selected). We interpret the failure of the structured procedure to counteract discrimination as due to the amount and complexity of the information that the participants had to process, but also that the participants were not motivated enough to make the required effort. Previous research has demonstrated that stereotypes exert greater influence when decisions are made in complex, information-loaded contexts [37, 16, 17]. Information load decreases decision quality [1, 38, 39, 40], increases the time required to make a decision, and increases confusion regarding the decision [41, 42]. Accordingly, discrimination against outgroup members was not counteracted by the structured procedure in study 1, where the job-applicants’ qualifications were more difficult to differentiate and the participants were likely overloaded with information. When in study 2, we put participants’ behavior under verbal stimulus control, informing them about a response cost if not involving themselves in the task enough, they were motivated to put more effort into the selection task. This increased the effect of the structured procedure and participants selected outgroup members even when dealing with the same number of résumés as in study 1, and confronted applicants who were difficult to distinguish with regard to qualifications. The effect of motivation to carry out the task carefully on selection outcome is a key finding of the current research, and points to the importance of sticking to the procedure when selecting personnel.

### Practical contributions

The main practical contribution of the current research concerns the experimental approach to investigating the effects of structured procedures on decision quality and discrimination in a selection context. The findings that an increased degree of structure enhances the ability to select competent applicants, lends clear support for the recommendations in *Principles for the validation and use of personnel selection procedures* from Society for Industrial and Organizational Psychology (SIOP, division 14 of the American Psychological Association), and ISO-standard 10667–1:2011 *Assessment service delivery—Procedures and methods to assess people in work and organizational settings*, which are broadly acknowledged guidelines on how to conduct an optimal selection process. Given that our studies provide (causal) experimental support of the effectiveness of these guidelines, they provide professional recruiters with even stronger reasons than before to employ structured and objective procedures.

As mentioned, there is consistent unanimity among researchers that structured recruitment is preferable [24], and the general recommendation is to use structured procedures and tools in all phases of the process. However, both previous research and the results from our study 1 suggest that tools per se may not be enough to counteract discrimination. Importantly, the finding from study 2 that a response-cost manipulation attenuated the somewhat careless approach that some of our participants took when conducting the job-analysis appear to have significant applied relevance in this regard. Of course, the motivation to work thoroughly, and thereby decrease the risk of discrimination, can be enhanced in other ways than with the approach we chose to use here and can be adapted to the context at hand. Organizations may instil routines to check performance intermittently, such that recruiters know that their work will be scrutinized, but not exactly when.

Another way to enhance the motivation to perform recruitment related tasks thoroughly is providing feedback to the recruiters during the recruitment process. For example, after having selected candidates, recruiters can be provided with information on how well they actually have performed, such as whether or not their selection decisions match their own previous ratings of the applicants. This way both recruiters and others are made aware of when and where the selection recommendations deviate in comparison to e.g. mechanically calculated scores.

Furthermore, organizations should follow evidence-based recommendations and procedures, for example prescribing the use of valid and reliable instruments, job-analyses and post-recruitment adjustments. Tools for fair recruitment will only have an impact if organizations actually use them. The legal system too plays an important role in prescribing fair measures, while at the same time increasing the possibilities for protected groups to press charges if they are discriminated against or if inadequate methods are used.

In sum, an implication of the motivation-related findings in study 2 is that organizations are well advised to ensure that recruiters adhere to the procedures, performing the tasks carefully and “by the book”. Our results suggest that it pays off to use structured procedures. The probability of selecting the most competent applicants is significantly higher. Further, undesirable effects of individual biases are reduced when recruiters are obliged to conduct a structured recruitment carefully. A perhaps even stronger incentive to adhere to structured procedures is legislation, prescribing the conduct of fair recruitment and selection processes.

### Limitations and recommendations for future research

Although providing important theoretical, empirical and practical contributions to the study of selection discrimination, there are some limitations to the performed studies that need to be taken into account. The main limitation relates to the external validity of the findings. All studies were conducted in a laboratory setting with convenience samples of university students as participants. This puts limits on the possibility to generalize the results to real selection situations on the labor market. It is important for future research to study the effects of increased structure in the population of professional recruiters too, preferably in real selection situations, to see how well the present results generalize. Since discrimination on the basis of ethnicity is a well-established fact in the modern labor market, it would appear that even professional recruiters need structured procedures in order to conduct a fair and unbiased selection. Many professional recruiters are reluctant to base their selection decision on tools and instead rely on their own personal impressions and judgements [22, 24, 25, 26, 27, 28].

Another limitation concerns the fact the present studies examined the effect of specific forms of structure. It is unclear whether adding further structured procedures would increase the effect of structure and be successful in contexts where the structured procedures employed in the present studies proved insufficient. Finally, the current research only concerns an early stage in the recruitment process, selection of applicants from a larger sample. The succeeding steps resulting in the final selection of a specific applicant (e.g. interview, testing, looking up references, etc; [43]) remain to be examined. It is thus important that future research examines the effect of different forms and varying degrees of procedural structure at different stages of the recruitment process. Despite these limitations, the findings are promising in that they provide support for the hypothesis that increased procedural structure in applicant selection improves the ability to identify the most competent applicants, while at the same time counteracting discriminatory behavior if they are used carefully and with high attention.

## Supporting information

S1 File SPSS data file providing the raw data of study 1. (SAV)

S2 File SPSS data file providing the raw data of study 2. (SAV)
